# Phenotypic and genotypic characterization of multidrug-resistant avian pathogenic *Escherichia coli* across life stages of captive African Houbara bustards: Conservation and One Health implications

**DOI:** 10.14202/vetworld.2026.654-666

**Published:** 2026-02-23

**Authors:** Sara M. Rashad

**Affiliations:** Labouratory manager, Bacteriology and Molecular biology units, International Foundation for Ecological Research (IFER), Errachidia, Morocco

**Keywords:** African Houbara bustard, antimicrobial resistance, avian pathogenic *Escherichia coli*, captive breeding, extended-spectrum beta-lactamase, multidrug resistance, One Health, virulence genes

## Abstract

**Background and Aim:**

The African Houbara bustard (*Chlamydotis undulata*), a species of high conservation concern, is maintained through intensive captive breeding programs where bacterial diseases may compromise productivity and reintroduction success. *Escherichia coli* is frequently implicated in reproductive failure, early mortality, and septicemic conditions; however, integrated phenotypic and genotypic characterization of antimicrobial resistance and virulence across life stages in this species remains scarce. This study aimed to determine the prevalence of *E. coli* across different life stages of captive African Houbara bustards, characterize multidrug-resistant (MDR), extensively drug-resistant (XDR), and extended-spectrum β-lactamase (ESBL) phenotypes, and profile avian pathogenic *E. coli* (APEC)–associated virulence genes within a One Health framework.

**Materials and Methods:**

A cross-sectional diagnostic investigation was conducted during the 2025 production season. A total of 110 samples were collected from early embryonic death (n = 50), chicks (n = 25), juveniles (n = 25), and adult females (n = 10). Isolation and identification of *E. coli* were performed using conventional bacteriology and the VITEK®2 system. Antimicrobial susceptibility testing was carried out using disk diffusion and VITEK®2 AST, with resistance patterns classified according to international MDR and XDR definitions. Phenotypic ESBL detection was undertaken, and a multiplex real-time polymerase chain reaction (PCR) assay targeting 15 APEC-associated virulence genes was applied to representative isolates.

**Results:**

*E. coli* was isolated from 62.7% (69/110) of samples, with the highest prevalence observed in juveniles (84%) and chicks (80%). All isolates were resistant to ampicillin and fluoroquinolones, while complete susceptibility was observed to fosfomycin, aminoglycosides, and chloramphenicol. MDR was detected in 96.9% of tested isolates, and one XDR isolate resistant to 15 antibiotics was identified. ESBL-producing *E. coli* were detected in juveniles (14%) and early embryonic death samples (9%). Virulence profiling revealed a high gene burden, with most isolates harboring ≥10 virulence genes, particularly those associated with iron acquisition, protectins, and invasion.

**Conclusion:**

Captive African Houbara bustards harbor highly virulent MDR and ESBL-producing *E. coli* across life stages, posing significant conservation, veterinary, and public health risks. Strengthened biosecurity, prudent antimicrobial stewardship, and integrated One Health surveillance are essential to improve breeding success and safeguard reintroduction programs.

## INTRODUCTION

Of the 3800 tropical avian species worldwide, a substantial proportion has been reported as endangered or threatened [1]. According to the International Union for Conservation of Nature (IUCN) [2], the African Houbara bustard (*Chlamydotis undulata*) is listed on the IUCN Red List. The decline in Houbara bustard populations is primarily attributed to overhunting and habitat loss, particularly because this species is a traditional game bird for Arab falconers. The conservation of these birds is therefore imperative, and captive breeding programs play a critical role in maintaining healthy populations, reinforcing wild stocks, and safeguarding against potential population collapse [2–5]. In 2013, a captive breeding program was established at the International Foundation for Ecological Research (IFER) in Errachidia, Morocco, with the objective of conserving and reintroducing this species into its natural habitat.

Several factors, including genital infections such as salpingitis, peritonitis, and salpingo-peritonitis, can adversely compromise the reproductive performance of females in captive breeding systems [6]. Similar conditions have been documented in poultry, with *Escherichia coli* being one of the most frequently isolated pathogens [7, 8]. Zoonotic and pathogenic bacteria have been reported in wild birds [9], including the Houbara bustard [10]. *E. coli* is a major etiological agent of omphalitis, yolk sac infection, and early mortality in young chicks [11–13]. Consequently, the emergence of antimicrobial-resistant bacteria represents one of the most significant challenges facing veterinary medicine and public health [14, 15]. The escalating threat of antibiotic resistance can substantially affect both sectors by restricting the effectiveness and availability of antimicrobial therapies [16]. Inappropriate and indiscriminate use of antibiotics is a key factor exacerbating this problem [17]. Notably, antibiotic-resistant bacteria have also been detected in wild animals inhabiting remote areas with minimal or no direct exposure to antimicrobial agents [18, 19]. The introduction of captive-bred birds into wild populations has been identified as a potential route for the dissemination of antibiotic-resistant bacteria [20, 21].

Since the 2000s, resistance among *Enterobacteriaceae* to third- and fourth-generation cephalosporins has increasingly been reported, largely due to the production of extended-spectrum β-lactamases, resulting in limited treatment options for affected infections [22–24]. Furthermore, the interaction between virulence factors and antibiotic resistance in *E. coli* influences pathogenicity, disease severity, bacterial survival, and therapeutic outcomes, thereby posing additional challenges for effective disease control [25, 26].

Despite the conservation importance of *C. undulata*, current knowledge on bacterial diseases affecting captive breeding populations remains fragmented and largely descriptive. Previous reports have primarily focused on clinical or pathological findings, with limited integration of phenotypic resistance profiles and genotypic virulence characteristics of *E. coli*. In particular, comprehensive investigations that simultaneously address multidrug-resistant (MDR), extensively drug-resistant (XDR), and extended-spectrum β-lactamase (ESBL) phenotypes, and APEC-associated virulence gene burdens across different life stages are lacking in conservation-managed bustards. Moreover, age-specific patterns of resistance and virulence, especially in early embryonic death, chicks, and juveniles, remain poorly understood, limiting inference on transmission dynamics within closed breeding systems. The absence of such integrative data represents a critical gap in conservation medicine, as it constrains evidence-based biosecurity planning, antimicrobial stewardship, and One Health risk assessment related to the release of captive-bred birds into wild populations.

The present study aimed to generate an integrated phenotypic and genotypic characterization of *E. coli* isolated from captive *C. undulata* across multiple life stages. Specifically, the objectives were to determine the prevalence of *E. coli* in early embryonic death, chicks, juveniles, and adult females; characterize MDR, XDR, and ESBL phenotypes; assess APEC-associated virulence gene profiles using multiplex real-time polymerase chain reaction (PCR); and explore potential transmission patterns within the captive breeding system. By addressing these objectives, the study seeks to provide baseline evidence to support targeted disease control, rational antimicrobial use, and One Health–oriented conservation strategies for endangered bustard populations.

## MATERIALS AND METHODS

### Ethical approval

All samples were obtained from routine diagnostic postmortem examinations conducted at the IFER, Errachidia, Morocco. No live birds were handled or experimentally manipulated. Adult females, chicks, and juveniles died naturally or were submitted for necropsy as part of standard veterinary investigations, while egg samples originated from routine incubation monitoring. As no additional interventions were performed, formal ethical approval was not required. Institutional management provided a written exemption, and all procedures complied with national regulations and institutional animal welfare guidelines.

### Study period and location

This study was conducted during the 2025 production season (encompassing the breeding and rearing cycle from approximately January to June, aligned with the species’ natural reproductive period under captive management) at the IFER captive breeding facility in Errachidia, Morocco. The facility is located in a semi-arid region (coordinates approximately 31.93°N, 4.42°W) and serves as a dedicated center for the conservation and reintroduction of the African Houbara bustard. All sampling, necropsies, and laboratory analyses were performed on-site or at affiliated laboratories within the IFER premises to ensure biosecurity and compliance with conservation protocols.

### Study design and sampling strategy

This study was designed as a cross-sectional diagnostic investigation conducted during the 2025 production season. A purposive sampling strategy was used, in which all cases meeting the inclusion criteria were included in the study. In total, 110 samples were collected and categorized into distinct stages of the reproductive and early life cycle of C. undulata: early embryonic death (n = 50), chicks (n = 25), juveniles (n = 25), and adult females (n = 10). The selected sample size reflected the range of age categories encountered and corresponded to routine diagnostic submissions received during the study period to support bacteriological and molecular analyses.

### Clinical examination and necropsy procedures

A total of 110 cases were examined, comprising 10 adult females, 25 chicks, 25 juveniles, and 50 early embryonic death samples. Among adult females, six cases of sudden death were recorded, while four additional birds exhibited lethargy and anorexia prior to death, including two cases complicated by dystocia and oviductal impaction. Of the chicks examined, 15 died suddenly, and 10 displayed clinical signs of anorexia and lethargy before death. In the juvenile group, 12 birds died suddenly without overt clinical signs, whereas 13 showed systemic illness characterized by ruffled feathers, marked lethargy, and anorexia. Fifty egg samples exhibiting early embryonic mortality at 5–7 days of incubation were also included.

Necropsies were performed by licensed veterinarians following established standard protocols [27]. Postmortem examination revealed macroscopic lesions consistent with *E. coli* septicemia, including fibrinous pericarditis, perihepatitis, airsacculitis, exudate accumulation, peritonitis, and visceral adhesions.

### Sample collection and processing

All samples were collected aseptically from internal organs, including the heart, liver, lung, and spleen, as well as oviduct and ovary tissues from adult females when indicated by clinical and pathological findings. Eggshell surfaces were decontaminated by immersion in 70% ethanol for 5–10 s, air-dried, aseptically cracked, and the internal contents pooled [28]. Samples were transported to the IFER laboratory in sterile stomacher bags under cooled conditions. Tissue samples were pooled and processed for bacterial screening and isolation.

### Bacteriological isolation and identification of *E. coli*

Isolation and identification of *E. coli* were performed following previously described methods [29]. Briefly, pooled organ samples were pre-enriched in peptone water and incubated aerobically at 37°C for 24 h, followed by streaking onto MacConkey agar and EMB agar (Oxoid, Hampshire, UK) and incubation at 37°C for 24–48 h. Presumptive colonies were subcultured to obtain pure isolates. Biochemical identification was carried out using the VITEK®2 system (bioMérieux SA, Marcy-l’Étoile, France). Bacterial suspensions were adjusted to 0.5 McFarland, loaded into VITEK®2 GN cards (Ref. No. 21341), and processed according to the manufacturer’s instructions to obtain species-level identification within the Enterobacteriaceae family.

### Antimicrobial susceptibility testing and ESBL phenotyping

Antimicrobial susceptibility testing was performed on a representative subset of 32 isolates using VITEK®2 GN97 cards (Ref. No. 42000, bioMérieux SA, Marcy-l’Étoile, France) to assess susceptibility to 18 antimicrobial agents and screen for ESBL production. The disk diffusion method was additionally applied following EUCAST guidelines and the latest VITEK®2 clinical breakpoint tables (version 14.0, 2024) [30]. *E. coli* ATCC 25922 was used as the quality control strain. Resistance patterns were classified according to international definitions [31] as MDR (resistance to ≥1 agent in ≥3 antimicrobial classes) and XDR (resistance to ≥1 agent in all but one or two classes).

### Genomic DNA extraction and quality assessment

Genomic DNA was extracted from 20 overnight bacterial cultures grown to an optical density of 1.0–1.2 at OD600. Cell pellets were obtained by centrifugation at 8,000 × *g* for 5 min and processed using the Kylt® DNA/RNA Kit (Kybio Co., Ltd., Shenzhen, China) following the manufacturer’s instructions. DNA concentration and purity were assessed using a NanoDrop spectrophotometer (Thermo Fisher Scientific, Waltham, MA, USA), with acceptable purity defined as an A260/A280 ratio of 1.8–2.0.

### Multiplex real-time PCR for APEC virulence genes

Virulence-associated genes were detected using the Kylt® APEC qPCR Kit (Kybio Co., Ltd., Shenzhen, China), targeting 15 APEC-related genes, including adhesins (papC, tsh), invasion (ibeA), iron acquisition (iucD, iutA, irp2, iroN), toxins (astA, vat, F11, hlyF), protectins (sitA, cvi/cva, iss), and outer membrane protease (ompT). Four multiplex reactions (APEC1–APEC4) were prepared in a final volume of 20 µL containing 16 µL master mix and 4 µL template DNA. Amplification was performed using a QuantStudio™ 5 Real-Time PCR System (Thermo Fisher Scientific, Waltham, MA, USA) with cycling conditions of 95°C for 10 min, followed by 42 cycles of 95°C for 15 s and 60°C for 60 s. Samples with Ct ≤ 30 were considered positive. Negative controls were included in all runs. Targeted genes are summarized in Table 1.

**Table 1:** Targeted virulence genes in four multiplex polymerase chain reaction (PCR) mixes for avian pathogenic *Escherichia coli*.

Real-time PCR mix	HEX (target gene)	FAM (target gene)	Cy5 (target gene)	TXR (target gene)
APEC1	Internal Control	*papC* gene	*tsh* gene	*irp2* gene
APEC2	*iss* gene	*iucD* gene	*F11* gene	*astA* gene
APEC3	*ibeA* gene	*vat* gene	*cvi/cva* gene	*iutA* gene
APEC4	*iroN* gene	*hlyF* gene	*ompT* gene	*sitA* gene

### Statistical analysis

Data were analyzed using Microsoft Excel® and SPSS® version 26 (IBM Corp., Armonk, NY, USA). Descriptive statistics were used to calculate frequencies and percentages of *E. coli* isolates and virulence genes. Fisher’s exact test was applied to compare prevalence among groups due to small sample sizes. Statistical significance was set at p ≤ 0.05. Confidence intervals are presented in Table 2.

**Table 2:** Prevalence of *Escherichia coli* isolates from different life stages of Houbara bustard.

Sample sources	Organs/tissue	Number of samples collected	No. of positive samples	% Positive (95% CI)	Interpretation
Dead females	Heart-liver-lung-spleen-oviduct	10	6	60 (29.6–90.4)	Small sample size is less precise
Egg (EED)	Egg content	50	22	44 (30.3–57.7)	Larger sample makes it more reliable
Chicks	Heart-liver-lung-spleen	25	20	80 (64.3–95.7)	Strong and clear results
Juvenile	Heart-liver-lung-spleen	25	21	84 (69.6–98.4)	Strong and clear results
Total	—	110	69	62.7 (53.7–71.7)	Large sample and precise result

EED = Early embryonic death, CI = Confidence interval.

## RESULTS

### Isolation and identification of *E. coli*

*E. coli* was isolated from 69 of 110 cases (62.7%). The highest isolation rate was observed in juveniles (84%), followed by chicks (80%), adult females (60%), and early embryonic death (44%). Sixty-two clinical isolates were identified as *E. coli* using the VITEK®2 system, with identification confidence ranging from 96% to 99%. Minor deviations in expected biochemical reactions (PHOS-81 and dTAG-22) were detected in a limited number of isolates; however, overall identification confidence remained high. Confidence intervals were calculated for each group.The prevalence of *E. coli* isolates across different life stages of *C. undulata* is summarized in Table 2.

### Phenotypic clustering and antimicrobial resistance profiles

**Figure 1:**
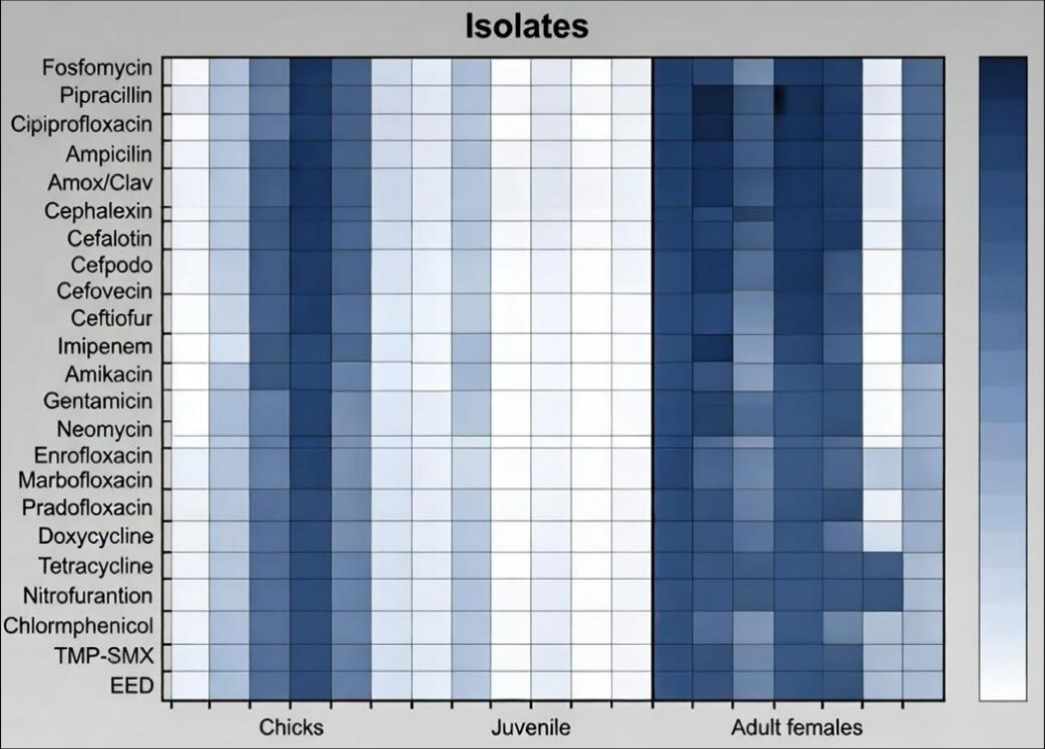
Heatmap showing the percentage resistance of *Escherichia coli* isolates to individual antibiotics, stratified by sample source (early embryonic death, chicks, juveniles, and adult females) in captive African Houbara bustards.

Two clustering patterns were identified: phenotypic clustering based on antimicrobial susceptibility profiles and genotypic clustering based on virulence gene distribution. Figure 1 presents a heat map illustrating resistance percentages among *E. coli* isolates recovered from early embryonic death, chicks, juveniles, and adult females.

Heatmap showing the percentage resistance of *Escherichia coli* isolates to individual antibiotics, stratified by sample source (early embryonic death, chicks, juveniles, and adult females) in captive African Houbara bustards.

Isolates originated from eggs (n = 8), chicks (n = 7), juveniles (n = 12), and adult females (n = 5). No resistance was detected to fosfomycin, amikacin, gentamicin, neomycin, or chloramphenicol. In contrast, all isolates exhibited resistance to enrofloxacin (ENR) and ampicillin (AMP).

### Distribution of multidrug-resistant phenotypes

**Figure 2:**
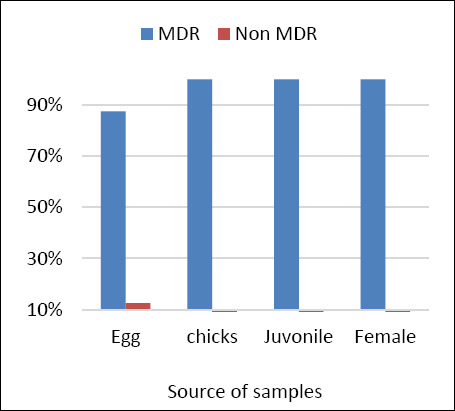
Distribution of multidrug-resistant (MDR) *Escherichia coli* isolates across sample groups (early embryonic death, chicks, juveniles, and adult females).

The distribution of phenotypic antibiotic resistance patterns is shown in Table 3. A total of 25 distinct multidrug-resistant (MDR) patterns were identified, ranging from resistance to 3 to 15 antibiotics. The most frequent resistance profile consisted of AMP, marbofloxacin (MAR), pradofloxacin (PRA), doxycycline (DOX), tetracycline (TET), nitrofurantoin (NIT), and trimethoprim–sulfamethoxazole (TMP-SMX) and was observed in three isolates. Several additional resistance profiles involved combinations of 8–10 antibiotics. One isolate demonstrated resistance to 15 antibiotics, indicating an extensively drug-resistant (XDR) phenotype. AMP, fluoroquinolones, TET, NIT, and TMP-SMX were the most frequently represented antimicrobial classes across resistance patterns. The distribution of MDR among sample groups is presented in Figure 2. Of the 32 *E. coli* isolates examined, 31 (96.9%) were classified as MDR. All isolates from chicks, juveniles, and adult females exhibited 100% MDR. Only one isolate from the early embryonic death group was non-MDR, resulting in an MDR prevalence of 87.5% in that group.

**Table 3:** Distribution of phenotypic patterns of antibiotic resistance in *Escherichia coli* among different Houbara bustard samples.

Pattern no.	Resistant pattern (antibiotics)	Number of isolates with pattern	Number of antibiotics in pattern
1	CIP + AMP + AMC	1	3
2	AMP + MAR + DOX + TET + NIT + TMP-SMX	1	6
3	AMP + NEO + MAR + DOX + NIT + TMP-SMX	1	6
4	CIP + AMP + MAR + DOX + TET + NIT	1	6
5	AMP + CFV + MAR + PRA + TET + NIT	1	6
6	AMP + MAR + PRA + DOX + TET + NIT + TMP-SMX	3	7
7	CIP + AMP + MAR + PRA + TET + NIT + TMP-SMX	1	7
8	AMP + AMC + MAR + PRA + DOX + TET + NIT + TMP-SMX	2	8
9	CIP + AMP + AMC + MAR + DOX + TET + NIT + TMP-SMX	2	8
10	CIP + AMP + AMC + MAR + PRA + DOX + TET + NIT	2	8
11	CIP + AMP + MAR + PRA + DOX + TET + NIT + TMP-SMX	1	8
12	CIP + AMP + AMC + CFV + MAR + PRA + DOX + TET + NIT	1	9
13	CIP + AMP + AMC + CFX + MAR + DOX + TET + NIT + TMP-SMX	1	9
14	CIP + AMP + AMC + CFX + MAR + PRA + DOX + TET + NIT	1	9
15	CIP + AMP + AMC + CPD + MAR + PRA + DOX + NIT + TMP-SMX	1	9
16	CIP + AMP + AMC + MAR + PRA + DOX + TET + NIT + TMP-SMX	1	9
17	PRL + AMP + AMC + MAR + PRA + DOX + TET + NIT + TMP-SMX	1	9
18	PRL + AMP + CPD + MAR + PRA + DOX + TET + NIT + TMP-SMX	1	9
19	PRL + CIP + AMP + AMC + MAR + PRA + DOX + TET + NIT	1	9
20	CIP + AMP + AMC + CFX + CPD + MAR + DOX + TET + NIT + TMP-SMX	2	10
21	CIP + AMP + AMC + CFX + CPD + MAR + PRA + DOX + TET + NIT	1	10
22	PRL + CIP + AMP + AMC + MAR + PRA + DOX + TET + NIT + TMP-SMX	2	10
23	AMP + AMC + CFX + CPD + CFV + MAR + PRA + DOX + TET + NIT + TMP-SMX	1	11
24	PRL + CIP + AMP + AMC + CPD + MAR + PRA + DOX + TET + NIT + TMP-SMX	1	11
25	CIP + AMP + AMC + CFX + CLT + CPD + CFV + CEF + IPM + MAR + PRA + DOX + TET + NIT + TMP-SMX	1	15

AMP = Ampicillin, AMC = Amoxicillin-Clavulanic Acid, PRL = Piperacillin, CIP = Ciprofloxacin, CLT = Cefalotin, CPD = Cefpodoxime, CFV = Cefovecin, CFX = Cefalexin, CEF = Ceftiofur, IPM = Imipenem, NEO = Neomycin, MAR = Marbofloxacin, PRA = Pradofloxacin, DOX = Doxycycline, TET = Tetracycline, NIT = Nitrofurantoin, TMP-SMX = Trimethoprim-Sulfamethoxazole.

Distribution of multidrug-resistant (MDR) *Escherichia coli* isolates across sample groups (early embryonic death, chicks, juveniles, and adult females).

### Life stage–specific MDR patterns

**Figure 3:**
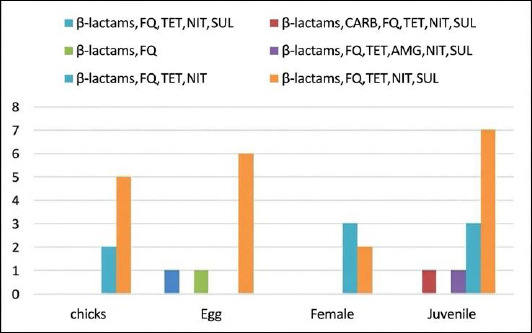
Distribution of multidrug-resistant patterns across life stages of Houbara bustards. β-lactams = Beta-lactams, FQ = Fluoroquinolones, TET = Tetracyclines, SUL = Sulfonamides, NIT = Nitrofurans, AMG = Aminoglycosides, CARB = Carbapenems.

Figure 3 illustrates the distribution and frequency of MDR patterns across life stages. The most predominant resistance combination consisted of β-lactams, fluoroquinolones, TET, NIT, and sulfonamides in chicks (71.5%) and eggs (75%). Adult females exhibited a higher proportion (60%) of resistance to β-lactams, fluoroquinolones, TET, and NIT. Juvenile isolates showed multiple resistance combinations. These findings indicate substantial variation in MDR patterns among life stages, although overall MDR prevalence remained consistently high (96.9%) across all groups.

### Statistical analysis of antimicrobial resistance

Statistical analysis of resistance rates by antimicrobial class is presented in Table 4. Due to small sample sizes in certain groups, Fisher’s exact test was applied instead of the chi-square test. No statistically significant differences in resistance rates were detected among early embryonic death, chicks, juveniles, and adult females (p > 0.05), largely due to uniform resistance and susceptibility patterns across most antimicrobial classes.

**Table 4:** Statistical analysis of antibiotic resistance by families across Houbara bustard groups using Fisher’s exact test and Chi-square test.

Antibiotic family	EED (n=8)	Chicks (n=7)	Juveniles (n=12)	Adult females (n=5)	Test used	p-value	Significance
Aminoglycosides	0%	0%	0%	0%	N/A (all sensitive)	N/A	NS
Fluoroquinolones	100%	100%	100%	100%	N/A (all resistant)	N/A	NS
Beta-lactams	100%	100%	100%	100%	N/A (all resistant)	N/A	NS
Carbapenem	0%	0%	0%	0%	N/A (all sensitive)	N/A	NS
Phosphonic acid	0%	0%	0%	0%	N/A (all sensitive)	N/A	NS
Tetracyclines	87.5%	100%	100%	100%	Fisher’s exact test	0.33	NS
Nitrofurans	87.5%	100%	100%	100%	Fisher’s exact test	0.33	NS
Phenicol	0%	0%	0%	0%	N/A (all sensitive)	N/A	NS
Folate pathway inhibitors	87.5%	71.4%	75%	40%	Fisher’s exact test	0.20	NS

EED = Early embryonic death, NS = Non-significant, N/A = Non-applicable.

### Prevalence of ESBL-producing *E. coli*

**Figure 4:**
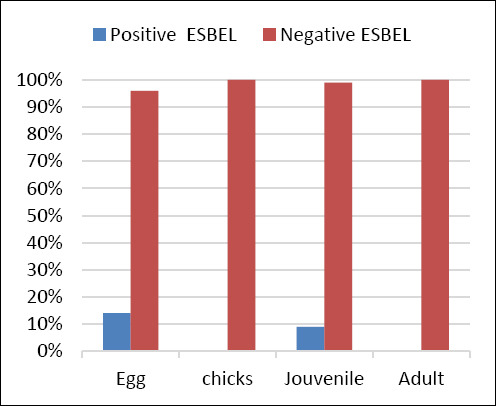
Prevalence of extended-spectrum β-lactamase-producing (ESBL) *Escherichia coli* in Houbara bustards across different life stages (early embryonic death, chicks, juveniles, and adult females).

The prevalence of extended-spectrum β-lactamase–producing *E. coli* across life stages is shown in Figure 4. ESBL-producing isolates were detected in 14% of juvenile samples and 9% of egg samples, indicating stage–specific occurrence and highlighting the need for targeted monitoring during early developmental stages.

### Virulence gene profiles of APEC isolates

The prevalence of avian pathogenic *E. coli*–associated virulence genes is summarized in Table 5. Most isolates harbored irp2, iss, iucD, iutA, ompT, and sitA, which were consistently detected across isolates from chicks, early embryonic death, juveniles, and adult females. In contrast, papC and tsh were absent in all isolates. Greater virulence gene diversity was observed in chick and egg isolates compared with juvenile isolates.

**Table 5:** Prevalence of APEC-associated 15 virulence genes in selected *Escherichia coli* isolates recovered from early embryonic death, chicks, juveniles, and adult female African Houbara bustards.

Isolate ID	Adhesins		Iron Acquisition					Toxins		Protectins				Invasins	
				
	*papC*	*tsh*	*irp2*	*iucD*	*iutA*	*sitA*	*iroN*	*astA*	*vat*	*F11*	*hlyF*	*iss*	*cvi/cva*	*ompT*	*ibeA*
Chick-1	–	–	+	+	+	+	+	–	+	–	+	+	+	+	+
Chick-2	–	–	+	+	+	+	+	–	+	–	+	+	+	+	+
Chick-3	–	–	+	+	+	+	+	–	–	+	+	+	+	+	–
Chick-4	–	–	+	+	+	+	+	–	+	–	+	+	+	+	+
Chick-5	–	–	+	+	+	+	–	–	–	–	+	+	+	+	–
EED-1	–	–	+	+	+	+	+	–	+	–	+	+	+	+	+
EED-2	–	–	+	+	+	+	+	+	+	–	+	+	+	+	+
EED-3	–	–	+	+	+	+	+	–	+	–	+	+	+	+	+
EED-4	–	–	+	+	+	+	+	+	+	–	+	+	+	+	+
EED-5	–	–	+	+	+	+	+	–	+	–	+	+	+	+	+
Female-1	–	–	+	+	+	+	+	–	–	+	+	+	–	+	–
Female-2	–	–	+	+	+	+	+	–	–	–	+	+	+	+	–
Female-3	–	–	+	+	+	+	+	–	+	–	+	+	+	+	+
Female-4	–	–	+	+	+	+	+	+	+	–	+	+	+	+	+
Female-5	–	–	+	+	+	+	+	–	+	–	+	+	+	+	+
Juvenile-1	–	–	–	–	–	+	+	–	–	–	+	+	+	+	–
Juvenile-2	–	–	–	+	+	+	+	–	–	–	+	+	+	+	–
Juvenile-3	–	–	+	+	–	+	+	–	+	–	+	+	+	+	+
Juvenile-4	–	–	+	–	–	+	+	–	+	–	+	+	+	+	+
Juvenile-5	–	–	–	+	+	+	+	–	–	–	+	+	+	+	–

EED = Early embryonic death APEC = Avian pathogenic *Escherichia coli* (+) = Presence of the gene (–) = Absence of the gene.

**Figure 5:**
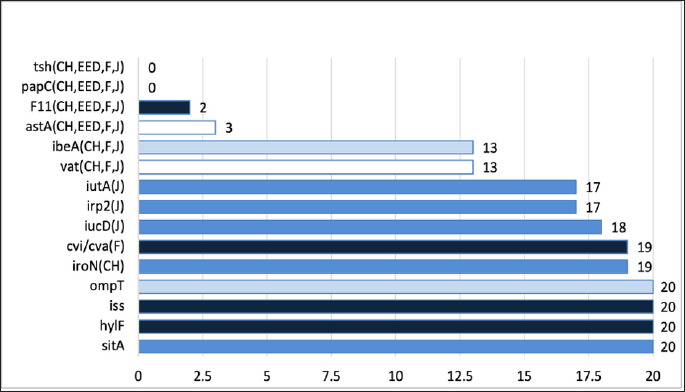
Gene prevalence and absence patterns for 15 avian pathogenic *Escherichia coli*-associated virulence genes among 20 selected *E. coli* isolates, grouped by source (n = 20). Prevalence numbers (x/20) = Total number of positive isolates (isolates possessing the gene). The presence of a group letter next to a gene indicates absence of that gene in the corresponding group (CH = chicks, EED = early embryonic death, F = adult female, and J = juvenile).

Gene prevalence and patterns of gene absence by isolate group (n = 20) are illustrated in Figure 5. A conserved core gene set, including protectin- and invasion-associated genes, was detected in 100% (20/20) of isolates, indicating their essential role in bacterial survival and pathogenicity. Iron acquisition genes exhibited the highest collective prevalence (17/20–20/20), emphasizing their importance in nutrient acquisition. Conversely, adhesin and toxin genes were detected at low frequencies (0/20–3/20), reflecting heterogeneity in pathogenic potential.

Gene prevalence and absence patterns for 15 avian pathogenic *Escherichia coli*-associated virulence genes among 20 selected *E. coli* isolates, grouped by source (n = 20). Prevalence numbers (x/20) = Total number of positive isolates (isolates possessing the gene). The presence of a group letter next to a gene indicates absence of that gene in the corresponding group (CH = chicks, EED = early embryonic death, F = adult female, and J = juvenile).

### Mean virulence gene burden across life stages

**Figure 6:**
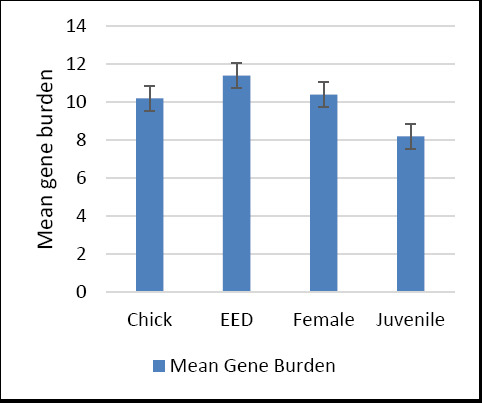
Mean number of virulence genes (gene burden) carried by *Escherichia coli* isolates across different life stages of Houbara bustards. EED = Early embryonic death.

The mean virulence gene burden across life stages of *C. undulata* is shown in Figure 6. Juvenile isolates exhibited the lowest standard deviation, indicating consistent gene burden values within this group, whereas chick isolates showed the highest standard deviation, reflecting greater variability in virulence gene distribution.

Mean number of virulence genes (gene burden) carried by *Escherichia coli* isolates across different life stages of Houbara bustards. EED = Early embryonic death.

## DISCUSSION

### Current knowledge gaps and study contribution

Only limited literature has addressed MDR *E. coli* and APEC-associated virulence factors in *C. undulata*, resulting in a substantial gap in understanding the pathogenic potential and epidemiology of *E. coli* in endangered avian species [8, 32, 33]. Consequently, the present findings represent one of the first comprehensive investigations integrating phenotypic MDR profiling with genotypic virulence characterization, including the detection of XDR isolates circulating within captive flocks [34, 35]. The combined phenotypic–genotypic framework applied in this study enabled detailed assessment of pathogen dynamics across life stages, supported by profiling of 15 virulence-associated genes [36, 37]. Isolates recovered from breeders, early embryonic death, chicks, and juveniles were associated with elevated mortality in captive African Houbara bustards (C. undulata).

### Prevalence of *E. coli* across life stages

The overall isolation rate of *E. coli* (62.7%) in *C. undulata* was notably high. This contrasts with a previous report describing 100% prevalence in postmortem samples from *Chlamydotis macqueenii* [38]. The highest isolation rates were recorded in juveniles (84%) and chicks (80%), supporting the association between immature immune function and age-related susceptibility in avian species. Environmental stressors have been widely reported as predisposing factors for colibacillosis in young birds [39–41]. Isolation rates in adult females (60%) during the breeding season were consistent with previous findings identifying *E. coli* as the predominant pathogen in clinical reproductive disorders of captive Houbara bustards [6]. Detection of *E. coli* in early embryonic death samples (44%) supports the hypothesis of horizontal or vertical transmission in avian systems [42–45], although recent studies indicate that true vertical transmission in poultry remains limited, with environmental contamination playing a more dominant role [46, 47].

### Antimicrobial resistance patterns and XDR emergence

Marked phenotypic resistance was observed against AMP, ENR, MAR, TET, NIT, DOX, and PRA across all isolates, in agreement with previous reports from Houbara bustards and poultry production systems [38, 42, 48, 49]. Earlier studies documented low ENR resistance in Houbara bustards and other wild birds [38, 50]; therefore, the widespread ENR resistance observed here indicates a concerning shift in resistance dynamics. Notably, PRA resistance was identified for the first time in Houbara bustards, highlighting the emergence of novel resistant phenotypes.

In contrast, complete susceptibility was observed to fosfomycin, amikacin, gentamicin, and chloramphenicol, supporting their potential role as alternative therapeutic options. These findings align with previous observations in poultry, where universal susceptibility to fosfomycin was reported [51]. Low resistance levels against cefalotin, cefovecin, ceftiofur, and neomycin in juvenile isolates further suggest limited but promising treatment alternatives for APEC infections in captive bustards.

Alarmingly, one isolate exhibited resistance to 15 antibiotics, including imipenem, indicating the emergence of XDR strains (Table 3). Similar findings have been reported in poultry populations [35]. MDR patterns were consistently distributed across all life stages, predominantly involving β-lactams, fluoroquinolones, TET, NIT, and sulfonamides (40%–75%), corroborating earlier reports linking resistance gene accumulation with treatment failure risks [32, 33].

### ESBL-producing *E. coli* and public health implications

The indiscriminate use of antibacterial agents across human, veterinary, and agricultural sectors has been implicated in the global dissemination of ESBL-producing bacteria [52, 53]. Sub-therapeutic antibiotic exposure in poultry has been proposed as a driver for environmental dissemination of ESBL-producing *E. coli*, posing substantial public health risks [22, 54]. While transmission of ESBL-producing *E. coli* between poultry products and humans has been documented [55], data regarding transmission from captive Houbara bustards to personnel remain unavailable.

In the present study, ESBL-producing *E. coli* were detected primarily during early life stages, with positivity rates of 15% in early embryonic death and 10% in juveniles. These findings are consistent with previous observations suggesting early horizontal exposure or breeder-associated transmission [36]. Juveniles, due to immunological immaturity, may facilitate persistence and dissemination of resistant strains [32, 33]. The absence of statistically significant differences among age groups (p > 0.05) supports flock-level dissemination of MDR *E. coli*, reinforcing the need for stringent biosecurity during incubation and early rearing.

### Virulence gene distribution and life stage specificity

APEC isolates are characterized by diverse virulence determinants, including adhesins, toxins, iron acquisition systems, and invasion-associated factors [56, 57]. Distinct life stage–specific virulence patterns were identified in captive C. undulata. Early embryonic death and chick isolates exhibited the highest virulence gene burdens, with most isolates harboring ≥10 genes, including iss, iucD, iutA, ompT, hlyF, and sitA. Similar gene burden–based pathogenicity classifications have been reported in poultry [58], although others have questioned the discriminatory power of virulence gene profiling alone [59].

Juvenile isolates displayed intermediate virulence profiles, potentially reflecting a transitional phase with reduced pathogenic potential. Adult female isolates retained core virulence genes, such as iss, iutA, and ompT, but exhibited lower overall gene richness, consistent with previous reports in Houbara bustards [6]. Reduced virulence gene prevalence may be associated with enhanced immune competence or cumulative environmental exposure.

The absence of certain adhesion-associated genes further emphasizes that *E. coli* pathogenicity is multifactorial and influenced not only by virulence gene content but also by host immunity, susceptibility, and environmental and management conditions [8, 33, 60, 61].

## CONCLUSION

This study demonstrated a high burden of *E. coli* infection (62.7%) across life stages of captive *C. undulata*, with the highest prevalence observed in juveniles and chicks. An alarmingly high proportion of isolates exhibited MDR (96.9%), with the detection of an XDR isolate confirming the emergence of highly resistant strains within the breeding system. ESBL-producing *E. coli* were predominantly detected during early life stages, supporting early acquisition and flock-level dissemination. Genotypic analysis revealed that most isolates carried multiple APEC-associated virulence genes, with early embryonic death and chick isolates harboring the highest gene burdens, particularly iss, iucD, iutA, ompT, hlyF, and sitA.

The coexistence of MDR, XDR, ESBL phenotypes, and high virulence gene loads in *E. coli* poses significant challenges for disease control, treatment efficacy, and conservation success in captive C. undulata. These findings highlight the urgent need for strengthened biosecurity, rational antimicrobial stewardship, and routine resistance surveillance within captive breeding facilities. Targeted hygiene interventions during incubation and early rearing are particularly critical to reduce early-life exposure. From a One Health perspective, the presence of highly resistant and virulent strains underscores potential risks to personnel, surrounding wildlife, and the environment.

Key strengths include the integrated phenotypic–genotypic approach, simultaneous assessment of MDR, XDR, ESBL, and APEC profiles, and life stage–specific analysis across the production cycle. The application of a 15-gene virulence panel enabled high-resolution characterization of pathogenic potential, providing one of the most comprehensive datasets currently available for *E. coli* in conservation-managed bustards.

The limited number of adult female samples may restrict extrapolation of findings for this group. In addition, reliance on targeted virulence profiling without whole-genome sequencing constrained deeper resolution of resistance mechanisms, plasmid structures, and transmission pathways. The focus on diagnostic cases may also overrepresent clinically severe infections.

Future studies should incorporate whole-genome sequencing to resolve resistance determinants, mobile genetic elements, and transmission dynamics within and beyond breeding facilities. Longitudinal monitoring, environmental sampling, and assessment of breeder-associated transmission routes are warranted. Evaluation of non-antibiotic interventions, including vaccination strategies and probiotic-based approaches, may offer sustainable alternatives for disease mitigation.

Overall, this study provides critical evidence that captive *C. undulata* populations can serve as reservoirs of MDR, XDR, ESBL-producing, and highly virulent *E. coli*. Integrating rigorous biosecurity, antimicrobial stewardship, and One Health–aligned surveillance is essential to safeguard conservation outcomes, animal health, and public health interfaces in captive breeding and reintroduction programs.

## DATA AVAILABILITY

All the generated data are included in the manuscript.

## AUTHOR’S CONTRIBUTIONS

The author solely contributed to the study’s conception and design, data collection, analysis and interpretation, manuscript writing, and final approval of the submitted version.

## COMPETING INTERESTS

The authors declare that they have no competing interests.

## PUBLISHER’S NOTE

Veterinary World remains neutral with regard to jurisdictional claims in the published institutional affiliations.

## References

[1] Díaz S, Fargione J, Chapin FI, Tilman D (2009). Biodiversity loss threatens human well-being. PLoS Biol.

[2] IUCN/SSC (2014). The IUCN Red List of Threatened Species:*Chlamydotis undulata*. International Union for Conservation of Nature.

[3] Ralls K, Meadows L (2001). Captive breeding programs and the conservation of endangered species. Conserv Biol.

[4] Conde DA, Colchero F, Guillén J (2011). Integrating life history and demography in conservation programs. Biol Conserv.

[5] Taylor G, Lacy R, Feistner A (2017). Captive breeding in conservation. Zoo Biol.

[6] Crispo E, Smith J, Thompson R (2025). Genital infections in captive birds:salpingitis and peritonitis. Avian Pathol.

[7] Landman W, Feberwee A, Mevius D (2013). Avian colibacillosis:pathogenesis and control strategies. Avian Pathol.

[8] Nolan L, Barnes H, Vaillancourt J (2020). Colibacillosis in poultry:new insights. Poult Sci.

[9] Dobbin G, Paul N, Gibb ZZ (2005). Zoonotic bacteria in wild birds. J Wildl Dis.

[10] Stievenart C, Mohammed H (2004). Health status of Houbara bustards in captivity. Int J Avian Sci.

[11] Effendi, Ramandinianto SC, Wibowo S, Fauziah I, Kusala MKJ, Fauzia KA, Furqoni AH, Raissa R (2024). Omphalitis and yolk sac infection in poultry:etiology, pathology, and epidemiology. Vet World.

[12] Hermawan FA, Nadania Zega DIS, Triatjaya Y, Khairani S, Pratiwi U (2024). Anatomical pathology features in day-old chicks with omphalitis. ARSHI Vet Lett.

[13] Shahjada F, Rahman M, Islam K (2017). Pathogenic *E. coli*in poultry production. J Vet Sci.

[14] OIE (2019). World Organisation for Animal Health:antimicrobial resistance report. OIE Publishing.

[15] Naghavi M, Vollset SE, Ikuta KS, Swetschinski LR (2024). Global burden of bacterial antimicrobial resistance 1990–2021:a systematic analysis with forecasts to 2050. Lancet.

[16] World Health Organization (2025). Global Antibiotic Resistance Surveillance Report 2025. https://www.who.int/publications/i/item/9789240116337.

[17] Pulingam T, Rahman A, Khan S (2022). Inappropriate antibiotic use in veterinary medicine. Antibiotics.

[18] Bartoloni A, Cutts F, Leoni S (2004). Antibiotic resistance in remote areas. Trop Med Int Health.

[19] Gilliver R, Bennett M, Begon M (1999). Antibiotic-resistant bacteria in wild animals. Environ Microbiol.

[20] Woodford N, Kock R (1991). Transmission of antibiotic-resistant bacteria. J Antimicrob Chemother.

[21] Woodford N (2000). Extended-spectrum beta-lactamases in Enterobacteriaceae. Clin Microbiol Infect.

[22] Poirel L, Madec J, Lupo A (2018). Resistance to cephalosporins via ESBLs in *Enterobacteriaceae*. Front Microbiol.

[23] Giufrè M, Accogli M, Cerquetti M (2021). ESBL-producing *Ecoli*in animals and humans. Antibiotics.

[24] Cardozo M, Furlan J, Souza R (2022). Extended-spectrum beta-lactamase producing *E. coli*:epidemiology and public health implications. J Glob Antimicrob Resist.

[25] Pan Y, Zhao F, Li X (2020). Interplay between virulence factors and antibiotic resistance in *Ecoli*. Front Microbiol.

[26] Sora V (2021). Extraintestinal pathogenic *Ecoli*:virulence gene profiles and antimicrobial resistance patterns. Microorganisms.

[27] MajóMasferrer N, Dolz Pascual R (2019). Atlas of Avian Necropsy:Macroscopic Diagnosis Sampling (Updated Edition).

[28] El Ftouhy FZ, Nassik S, Nacer S, Kadiri A, Charrat N, Attrassi K, Hmyene A (2022). Bacteriological quality of table eggs in Moroccan formal and informal sector. Int J Food Sci.

[29] Nolan L, Barnes H, Vaillancourt J, Abdul-Aziz T, Logue CM, Saif YM, Fadly AM, Glisson JR, McDougald LR, Nolan LK, Swayne DE (2013). Colibacillosis. Diseases of Poultry.

[30] WHO Surveillance (2003). Antimicrobial Resistance:Global Report on Surveillance. https://iris.who.int/handle/10665/42789.

[31] Magiorakos AP, Srinivasan A, Carey RB (2012). Multidrug-resistant, extensively drug-resistant and pandrug-resistant bacteria:standard definitions for acquired resistance. Clin Microbiol Infect.

[32] Ewers C, Janssen T, Kiessling S, Philipp H, Wieler L (2007). Avian pathogenic *Escherichia coli* (APEC). Vet Microbiol.

[33] Dziva F, Stevens M (2008). Colibacillosis in poultry:pathogenesis and strategies for control. Avian Pathol.

[34] Liang X, Wu J, Zhang Q (2023). Multidrug-resistant *Ecoli*in poultry farms. Front Vet Sci.

[35] Aworh M, Kwaga J, Okeke I (2021). Emergence of extensively drug-resistant *Ecoli*in Nigerian poultry. Antimicrob Resist Infect Control.

[36] Beatrice S, Marco P, Elisa R (2025). Virulence genes and multidrug resistance in avian *Ecoli*. Vet Microbiol.

[37] Rafiq S, Khan A, Javed H (2024). Antibiotic resistance and virulence profiling in poultry *Ecoli*. Int J Vet Sci.

[38] Shobrak M, Hassan S, Stiévenart C, El-Deeb B, Gherbawy Y (2013). Prevalence and antibiotic resistance profile of intestinal bacteria isolated from captive adult Houbara bustards (Chlamydotis macqueenii) exposed to natural weather conditions in Saudi Arabia. Glob Veterinaria.

[39] Huff W, Rath N, Balog J (2015). Environmental stressors and colibacillosis in poultry. Poult Sci.

[40] El-Gazzar M, Kang S (2024). Stress and immune function in captive birds. J Avian Med Surg.

[41] Gedeno J, Otiang E, Makau D (2022). Risk factors for *Ecoli*infections in juvenile poultry. Vet Rec.

[42] Joseph J, Jennings M, Barbieri N, Zhang L, Adhikari P, Ramachandran R (2023). Characterization of avian pathogenic *Escherichia coli* isolated from broiler breeders with colibacillosis in Mississippi. Poultry.

[43] Shterzer N, Rothschild N, Sbehat Y, Dayan J, Eytan D, Uni Z (2023). Vertical transmission of gut bacteria in commercial chickens is limited. Anim Microbiome.

[44] Risalvato J, Sewid A, Eda S, Gerhold R, Wu J (2025). Strategic detection of *Escherichia coli* in the poultry industry:food safety challenges, One Health approaches, and advances in biosensor technologies. Biosensors.

[45] Tilli G, Rossi L, Bianchi A, Conti P (2024). A systematic review on the role of biosecurity to prevent or control colibacillosis in broiler production. Poult Sci.

[46] Long JR, Moore SJ, Woodward MJ (2022). Longitudinal study on background lesions in broiler breeder flocks and their progeny, and genomic characterization of *Escherichia coli*. Vet Res.

[47] Rychlik I (2023). Vertical transmission of gut bacteria in commercial chickens is limited. Anim Microbiome.

[48] Ibrahim G, Salah-Eldein A, Al-Zaban M, El-Oksh A, Ahmed E, Farid D (2023). Monitoring the genetic variation of some *Escherichia coli* strains in wild birds and cattle. Onderstepoort J Vet Res.

[49] Liao M, Wu J, Li Y, Lu X, Tan H, Chen S (2023). Prevalence and persistence of ceftiofur-resistant *Escherichia coli* in a chicken layer breeding program. Animals.

[50] Ahmed N, Gulhan T (2024). Determination of antibiotic resistance patterns and genotypes of *Escherichia coli* isolated from wild birds. Microbiome.

[51] Osman K, Kappell A, Elhadidy M, ElMougy F, Abd El-Ghany W, Orabi A (2018). Poultry hatcheries as potential reservoirs for antimicrobial-resistant *Escherichia coli*:a risk to public health and food safety. Sci Rep.

[52] Hosuru subramanya S, Bairy I, Nayak N, Padukone S, Sathian B, Gokhale S (2019). Low rate of gut colonization by extended spectrum β-lactamase producing *Enterobacteriaceae* in HIV infected persons as compared to healthy individuals in Nepal. PLoS ONE.

[53] Yang Y, Ashworth A, Willett C, Cook K, Upadhyay A, Owens P (2019). Review of antibiotic resistance, ecology, dissemination, and mitigation in U. S. broiler poultry systems. Front Microbiol.

[54] Rousham E, Unicomb L, Islam M (2018). Human, animal and environmental contributors to antibiotic resistance in low-resource settings:integrating behavioural, epidemiological and One Health approaches. Proc R Soc B Biol Sci.

[55] Falgenhauer L, Imirzalioglu C, Oppong K, Akenten C, Hogan B, Krumkamp R (2019). Detection and characterization of ESBL-producing *Escherichia coli* from humans and poultry in Ghana. Front Microbiol.

[56] Borzi M, Sordi M, Barbieri R (2018). Synergistic action of virulence factors in avian *Ecoli*. Vet Microbiol.

[57] Amer M, Mekky H, Amer A, Fedawy H (2018). Antimicrobial resistance genes in pathogenic *Escherichia coli* isolated from diseased broiler chickens in Egypt and their relationship with the phenotypic resistance characteristics. Vet World.

[58] Wang J, Tang P, Tan D, Wang L, Zhang S, Qiu Y (2015). The pathogenicity of chicken pathogenic *Escherichia coli* is associated with the numbers and combination patterns of virulence associated genes. Open J Vet Med.

[59] Sadek D, Rady M, Fedawy H, Rabie N (2020). Molecular epidemiology and sequencing of avian pathogenic *Escherichia coli* (APEC) in Egypt. Adv Anim Vet Sci.

[60] Collingwood C, Kemmett K, Williams N, Wigley P (2014). Is the concept of avian pathogenic *Escherichia coli* as a single pathotype fundamentally flawed?. Front Vet Sci.

[61] Heidemann Olsen R, Thøfner I, Pors S, Pires dos Santos T, Christensen J (2016). Experimental induced avian *Escherichia coli* salpingitis:significant impact of strain and host factors on the clinical and pathological outcome. Vet Microbiol.

